# Applying Market Basket Analysis to Determine Complex Coassociations Among Food Allergens in Children With Food Protein-Induced Enterocolitis Syndrome (FPIES)

**DOI:** 10.1177/23333928241264020

**Published:** 2024-07-25

**Authors:** Ankona Banerjee, Kenneth Nobleza, Cynthia Haddad, Joshua Eubanks, Ruchit Rana, Nicholas L. Rider, Lisa Pompeii, Duc Nguyen, Sara Anvari

**Affiliations:** 1 Division of Epidemiology, Department of Pediatrics, 3989 Baylor College of Medicine, Houston, TX, USA; 2 Department of Pediatrics, 3989 Baylor College of Medicine, Houston, TX, USA; 3 Division of Immunology, Allergy and Retrovirology, Texas Children's Hospital, 3989 Baylor College of Medicine, Houston, TX, USA; 4 Department of Health System & Implementation Science, Virginia Tech Carilion School of Medicine, Roanoke, VA, USA; 5 Division of Patient Services Research, Department of Pediatrics, 2518 Cincinnati Children's Hospital Medical Center, Cincinnati, OH, USA; 6 568693 Texas Children's Hospital, William T. Shearer Center for Human Immunobiology, Houston, TX, USA

**Keywords:** market basket analysis, epidemiology, food allergens

## Abstract

**Background:**

Food protein-induced enterocolitis syndrome (FPIES) is a non-IgE-mediated food allergy, characterized by delayed onset of repetitive vomiting occurring 1 to 4 h following ingestion of a food allergen. Managing FPIES requires strict avoidance of the food trigger. The concern with FPIES is determining the risk of another FPIES food trigger reaction due to potential coassociations with other foods or food groups. An effective statistical approach for analyzing FPIES-related data is essential to identify common coallergens and their associations.

**Methods:**

This study employed Market Basket Analysis, a data-mining technique, to examine correlations and patterns among allergens in FPIES patients at a Houston, Texas, pediatric tertiary center. A retrospective analysis of electronic medical records from January 2018 to March 2022 for allergist diagnosed FPIES patients was conducted. The analysis utilized R software, specifically the “arules” and “arulesViz” packages, implementing the Apriori algorithm with set minimum support and confidence thresholds.

**Results:**

The study included 210 FPIES cases over 4 years, with 112 patients reacting to one food trigger and 98 to more than one trigger. In the latter group, the 5 predominant triggers were cow's milk (45.9%), rice (31.6%), oats (30.6%), soy (22.4%), and avocado (19.4%). Market Basket Analysis identified significant associations between food categories, particularly between soy and dairy, egg and dairy, oat and dairy, rice and dairy, and avocado and dairy.

**Conclusion:**

Market Basket Analysis proved effective in identifying patterns and associations in FPIES data. These insights are crucial for healthcare providers in formulating dietary recommendations for FPIES patients. This approach potentially enhances guidance on food introductions and avoidances, thereby improving management and the quality of life for those affected by FPIES.

## Introduction

Food protein-induced enterocolitis syndrome (FPIES) is a non-IgE cell-mediated food allergy that presents with delayed severe vomiting 1 to 4 h after consuming a specific food trigger.^1^ Managing FPIES presents numerous challenges for patients, healthcare providers, and families, which can affect the patient's overall quality of life. One major challenge is the strict dietary avoidance of the food trigger(s).^2^ Compliance with this restriction can be challenging for patients and their families, especially when they need to avoid multiple allergens or when allergen-free alternatives are limited. The absence of allergen-free alternatives can further complicate the issue, as patients might struggle to find suitable food options, leading to an increased risk of nutritional deficiencies.^3^ Another challenge faced by healthcare providers is the complexity of diagnosing FPIES as the pathophysiology remains unknown therefore there are no diagnostic tests available.^2^ This can result in a delayed diagnosis, potentially prolonging patient suffering and increasing healthcare costs. The impact of FPIES on the quality of life for patients and their families is considerable. Parents may experience increased stress and anxiety due to the constant vigilance required to avoid food trigger(s) and the fear of potential reactions.^4^

There is an urgent need to identify coassociations between different foods in patients with diagnosed with FPIES, and to monitor the growth and nutritional status of children with FPIES^1^ However, statistical methods have limitations in identifying the food trigger(s) and the patterns of FPIES given the vast number of potential allergens and the complex interactions among different food groups. Therefore, it is critical to develop an effective statistical approach for analyzing FPIES-related data.

Market Basket Analysis (MBA) is a data-mining technique that aims to uncover associations between items or products by analyzing transactional data. Originally used in the business field, this method seeks to identify patterns of items frequently bought together, helping businesses understand the relationships among the products they sell. Market Basket Analysis provides insights into customer purchasing behavior, enabling organizations to make informed decisions about product placement, pricing, promotions, and recommendations to enhance customer satisfaction and increase sales.^5^

In one of the first studies that used MBA in clinical research, Wright et al suggested that it could be a useful data mining tool to identify associations between medications, laboratory results, and patient conditions.^6^ More recently, MBA has been increasingly utilized in various healthcare research studies, demonstrating its potential to reveal valuable insights into disease and treatment patterns. In a specific study, Rao et al applied MBA to analyze electronic health record data collected from 10 countries, involving 11 000 patients and 1000 diseases. Using the Apriori algorithm, they identified associations between different diseases across countries and regions, visualizing the results through heat maps and network graphs.^7^

These studies highlight the versatility of MBA as a tool for identifying patterns and associations in healthcare data, providing valuable insights for healthcare providers and researchers to improve patient care and treatment strategies. In this study, we aimed to evaluate the usefulness of MBA in exploring the correlations and patterns between different FPIES food triggers at a pediatric tertiary center.

## Methods

### Data Collection

We conducted a retrospective review of electronic medical records in EPIC for pediatric patients aged 0 to 17 years diagnosed with FPIES, who visited the Allergy/Immunology Clinic at Texas Children's Hospital during a 4-year period from January 2018 to March 2022. Food protein-induced enterocolitis syndrome patients were identified using the *ICD-10* code K52.21. Charts were reviewed by an allergist (SA). The study was approved by Baylor College of Medicine Institutional Review Board (H-53109).

### Participant Selection

The study design and participant selection have been described elsewhere.^8^ Briefly, the electronic records were examined to ensure that symptoms aligned with an FPIES diagnosis based on the International Consensus Guidelines on FPIES.^1^ Patients with an FPIES diagnosis code who met the clinical criteria according to provider documentation were included in the analysis. In total, 210 FPIES patients were part of this descriptive analysis. Data collected included race, ethnicity, sex, birth history, health insurance provider, infant feeding method, family history of atopy, atopic comorbidities, age of first episode, age at diagnosis, number of trigger foods, positive skin prick test, and/or specific IgE test results for the trigger food, clinical presentation, initial diagnoses, emergency department visits, hospitalization rates, medical interventions provided, dietetic consultation, abnormal laboratory findings, oral challenges performed, and trigger resolution.

## Data Analysis

*Data preparation and statistical packages:* The MBA analysis was conducted using the R software with the “arules” and “arulesViz” packages.^9–11^ Data cleaning was performed prior to the analysis during which individuals with only one food allergen were excluded. Differences between groups were compared using the χ^2^ or Fisher exact tests for categorical variables and Mann-Whitney *U* test for continuous variables as appropriate.

### Market Basket Analysis

Market Basket Analysis was conducted using the Apriori algorithm, a popular and efficient method for mining the frequent collections of items that occur together in a dataset (called “itemsets”) and generating association rules. The algorithm works by iteratively identifying the most frequent itemsets of a given size (k) that meet a predefined minimum support threshold, and then generating association rules for these itemsets using a minimum confidence threshold. For this study, itemsets are combinations of food allergens seen in an individual.

For our analysis, we set the minimum support threshold at 0.01 (representing 1% of transactions) and the minimum confidence threshold at 0.1 (indicating a 10% probability that the consequent allergen is associated with a higher risk of FPIES when the antecedent item is present).

To perform the MBA, we used the “arules” package in R, specifically designed for mining association rules and frequent itemsets. The analysis focused on identifying strong associations between food categories relevant to food allergies and detecting potentially hazardous food combinations for FPIES patients.

In MBA, “lift” is a metric used to measure the strength of the association between 2 items in a transaction dataset. It is derived from the concept of support, confidence, and association rules in data mining and is used to discover interesting relationships between itemsets. In our study, it helps determine the likelihood that 2 food items are to be coallergens.

Lift is calculated using the following formula:

Lift(A=>B)=P(AandB)/(P(A)*P(B))

where

- A and B are the food items being analyzed.
- P(A and B) is the probability that A and B occur together as coallergens.
- P(A) is the probability that A is an allergen.
- P(B) is the probability that B is an allergen.

Lift values can be interpreted as follows:

- Lift = 1: A and B are independent, and their dual presence has no effect on each other.
- Lift > 1: A and B have a positive association, meaning that the presence of one food item increases the likelihood of the other item being a coallergen.
- Lift < 1: A and B have a negative association, meaning that the presence of one food item decreases the likelihood of the other item being a coallergen.

By analyzing the lift values, allergists can gain insights into the relationships between food items and use this information to make more informed decisions about coallergens and risk of allergy between food groups.

## Results

### Demographics and Characteristics

We identified 210 cases of FPIES in a 4-year period with a median age of 6 months (IQR in months 4, 8). Males comprised 54.3% of the total participants. In this demographic, 53.3% (n = 112) were in the one trigger group, and 46.6% (n = 98) were in the more than one trigger group. Of the overall participants, 6.2% were Asian, 8.1% were Black, 73.8% were White, Native Hawaiian and Pacific Islander (0.5%), multiple races (0.5%), missing (0.5%), and 10.5% preferred not to answer. For the group with one trigger, 8.9% were Asians while 3.1% of the group with more than one triggers were Asians. Most participants identified as White both for the group with one trigger (67%) and for the group with more than one trigger (81.6%). Those who preferred not to answer the question on race were 10.5% for the group with one trigger and 14.3% for the group with more than one trigger; 8.9% of the group with one trigger were Black compared to 7.1% of the group with more than one trigger. The overall distribution of race showed a significant difference between the 2 groups (Fisher exact test, *P* = .043). Hispanics patients represented 21.4% of the study population. Of the Hispanic population, 23.2% were in the one trigger group and 19.4% were in the more than one trigger group. The overall distribution of ethnicity was not significantly different between the 2 groups (*P* = .43) (Table 1).

**Table 1.** Demographic Details of the Population Stratified by Number of Triggers.

Variable	Overall	One trigger	More than one trigger	*P* value
n	210	112	98	
Age at first episode in months, median (IQR)	6 (4-8)	6.25 (4-10.5)	6 (4-7)	.02
Age of first episode				.13
0-3 months	34 (16.2)	13 (11.6)	21 (21.4)	
4-5 months	41 (19.5)	19 (17.0)	22 (22.4)	
6-12 months	104 (49.5)	57 (50.9)	47 (48.0)	
13-18 months	10 (4.8)	6 (5.4)	4 (4.1)	
19-24 months	11 (5.2)	9 (8.0)	2 (2.0)	
25-30 months	1 (0.5)	1 (0.9)	0 (0.0)	
31-36 months	2 (1.0)	2 (1.8)	0 (0.0)	
37-42 months	0 (0.0)	0 (0.0)	0 (0.0)	
43-48 months	1 (0.5)	1 (0.9)	0 (0.0)	
>49 months	4 (1.9)	2 (1.8)	2 (2.0)	
Missing	2 (1.0)	2 (1.8)	0 (0.0)	
Age at first diagnosis in months, median (IQR)	9 (7-16.75)	11 (7-19)	8 (7-11)	<.01
Age of first diagnosis				.27
0-3 months	7 (3.3)	4 (3.6)	3 (3.1)	
4-5 months	8 (3.8)	5 (4.5)	3 (3.1)	
6-12 months	106 (50.5)	48 (42.9)	58 (59.2)	
13-18 months	29 (13.8)	20 (17.9)	9 (9.2)	
19-24 months	21 (10.0)	13 (11.6)	8 (8.2)	
25-30 months	9 (4.3)	4 (3.6)	5 (5.1)	
31-36 months	5 (2.4)	2 (1.8)	3 (3.1)	
37-42 months	5 (2.4)	3 (2.7)	2 (2.0)	
43-48 months	5 (2.4)	4 (3.6)	1 (1.0)	
>49 months	13 (6.2)	9 (8.0)	4 (4.1)	
Missing	2 (1.0)	0 (0.0)	2 (2.0)	
Prefer not to answer	22 (10.5)	16 (14.3)	6 (6.1)	
Missing	1 (0.5)	0 (0.0)	1 (1.0)	
Biological Sex (%)				.13
Male	114 (54.3)	55 (49.1)	59 (60.2)	
Female	96 (45.7)	57 (50.9)	39 (39.8)	
Race (%)				.04
Asian	13 (6.2)	10 (8.9)	3 (3.1)	
Black/African American	17 (8.1)	10 (8.9)	7 (7.1)	
White	155 (73.8)	75 (67.0)	80 (81.6)	
American Indian or Alaska Native	0 (0.0)	0 (0.0)	0 (0.0)	
Native Hawaiian or other Pacific Islander	1 (0.5)	0 (0.0)	1 (1.0)	
Multiple/Other	1 (0.5)	1 (0.9)	0 (0.0)	
Prefer not to answer	22 (10.5)	16 (14.3)	6 (6.1)	
Missing	1 (0.5)	0 (0.0)	1 (1.0)	
Ethnicity (%)				.43
Hispanic	45 (21.4)	26 (23.2)	19 (19.4)	
Non-Hispanic	150 (71.4)	76 (67.9)	74 (75.5)	
Unknown	15 (7.1)	10 (8.9)	5 (5.1)	
Insurance (%)				.50
Private	163 (77.6)	89 (79.5)	74 (75.5)	
Medicaid	39 (18.6)	18 (16.1)	21 (21.4)	
Military/ Veterans	5 (2.4)	3 (2.7)	2 (2.0)	
Uninsured	1 (0.5)	0 (0.0)	1 (1.0)	
Missing	2 (1.0)	2 (1.8)	0 (0.0)	
No. of triggers(n)				NA
1		112	0	
2		0	56 (57.1%)	
3		0	20 (20.4%)	
4		0	7 (7.1%)	
5		0	7 (7.1%)	
6		0	2 (2.0%)	
7		0	4 (4.1%)	
8		0	1 (1.0%)	
9		0	1 (1.0%)	

#### Triggers

For the group with more than one FPIES trigger, the 5 most common triggers were cow's milk seen in 45 individuals (45.9%), rice seen in 31 individuals (31.6%), oats observed in 30 individuals (30.6%), soy observed in 22 individuals (22.4%), and avocado (19.4%). For the group with one FPIES trigger, the 5 most common triggers were cow's milk seen in 29 (25.9%) individuals, followed by egg in 18 (16.1%) individuals, peanut in 13 (11.6%) individuals, oats in 10 (8.9%) individuals, and root vegetables (potato, sweet potato, and yam) in 9 (8%) individuals. Table 2 demonstrates differences observed between the 2 groups stratified by number of FPIES triggers. Cow's milk was a trigger in 45.9% of individuals with more than one triggers compared to 25.9% of those with one trigger (*P* < .01). Oat was an FPIES trigger in 8.9% in the group with one FPIES trigger and 30.6% in the group with more than one FPIES trigger, showing a significant difference (*P* < .001). Only 0.9% in the group with one FPIES trigger reported soy as a FPIES trigger, whereas it was a trigger for 22.4% in the more than one trigger group, indicating a significant difference (*P* < .001). None of the participants in the group mentioned corn as the sole FPIES trigger, while 6.1% in the group with more than one trigger did. This is statistically significant (*P* = .009).

**Table 2.** Differences in Food Triggers Between the Group With One FPIES Trigger Versus More Than One FPIES Trigger for the 6 Most Common Food Items.

Variable	Total, n (%)(95% CI)	One trigger, n (%)(95% CI)	More than one triggers, n (%)(95% CI)	*P*
*n*	210	112	98	
Oat (%)	40 (19.0) (14.1-25.2)	10 (8.9) (4.6-16.2)	30 (30.6) (21.9-40.9)	<.001
Rice (%)	36 (17.1) (12.4-23.1)	5 (4.5) (1.7-10.6)	31 (31.6) (22.8-41.9)	<.001
Soy (%)	23 (11.0) (7.2-16.2)	1 (0.9) (0.04-5.6)	22 (22.4) (14.9-32.2)	<.001
Cow's milk	74 (35.2) (28.9-42.1)	29 (25.9) (18.3-32.2)	45 (45.9) (35.9-56.3)	.004
Wheat (%)	10 (4.8) (2.4-8.8)	4 (3.6) (1.2-9.4)	6 (6.1) (2.5-13.4)	.52
Egg	37 (17.6) (12.9-23.6)	18 (16.1) (10.1-24.5)	19 (19.4) (12.4-28.9)	.59

Data are presented as the number of occurrences (percentage) and 95% confidence interval for each food item. See Supplemental Table 1 for this information for every food item.

### Multiple Food Allergens

#### Count of allergens

The majority of FPIES patients in the study had only one trigger (53.3%, n* *= 112), while 36.1% (n* *= 76) had 2 to 3 triggers and 10.5% (n* *= 22) had more than 3 triggers (Table 1).

In the entire study cohort (n* *= 210), FPIES to cow's milk alone was observed in 29 individuals, egg FPIES was observed in 18 individuals, and peanut FPIES was identified in 13 individuals. When combinations were identified, a combination of dairy and soy were observed in 9 individuals. Oat and rice FPIES were identified in 6 individuals and avocado and banana FPIES were identified in 3 individuals (Table 2 and Supplemental Table 1).

**Figure 1.**
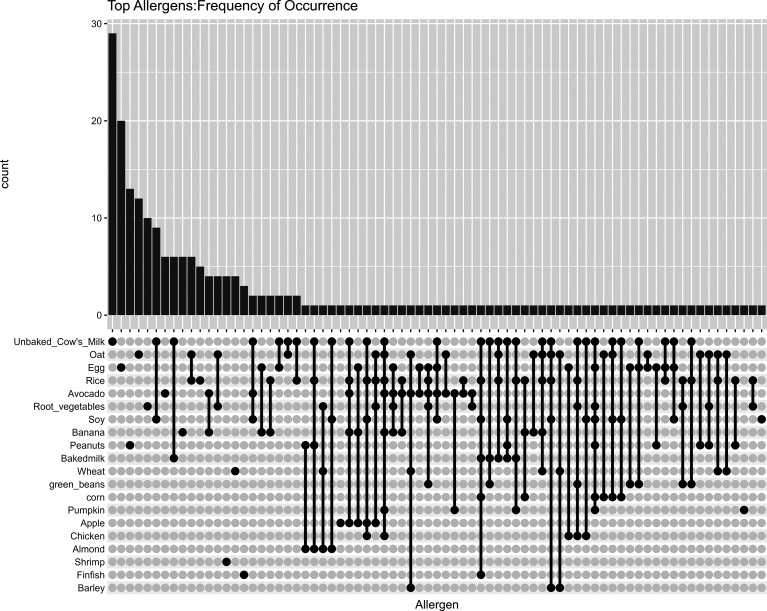
Visualization of allergen occurrence among participants, based on the frequency of each allergen.

In the study's visualization of allergen occurrence among participants, the upset plot organizes the data based on the frequency of each allergen. This provides an overview of the most common allergens across the cohort. The top 20 allergens, in terms of frequency, are represented in this plot (Figure 1). If a viewer wishes to observe when unbaked cow's milk and soy were the only 2 coallergens, the sixth bar graph has that information. If the viewer wishes to observe when unbaked cow's milk, soy milk, and avocado were the coallergens, the viewer needs to look at the 17th bar graph. Similarly, numbers of sole egg allergies are represented by the second bar graph.

**Figure 2.**
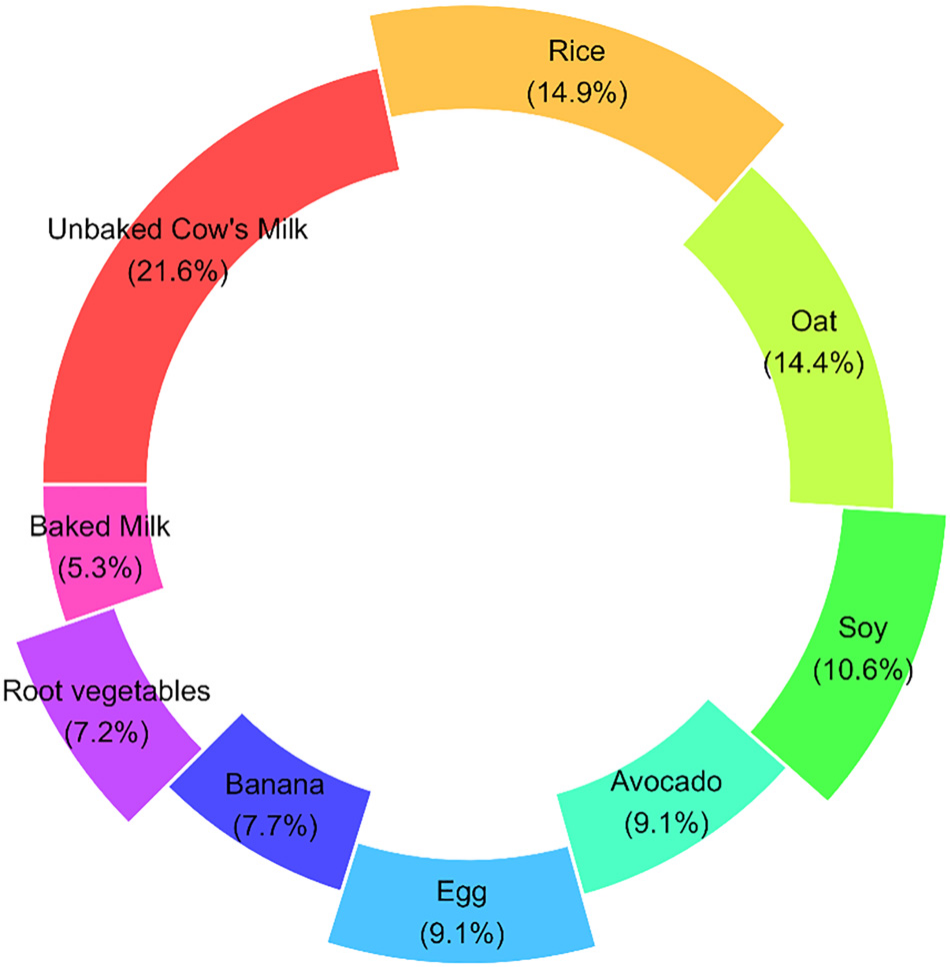
Distribution of triggers in those reporting FPIES to more than one food. The offset slices are intended to highlight the most common triggers.

It should be noted that 98 individuals in our study reported FPIES reaction to more than one allergen. This donut chart explains the distribution of triggers in those individuals (Figure 2). Like the group with just one trigger, the most common trigger in the group with more than one trigger was cow's milk (21.6%). In the group with one trigger, the second most common trigger was egg (16.1%) while in the group with more than one trigger the second most common trigger was rice (31.6%).

#### Market basket analysis

As simple descriptives may not be the ideal way to understand the complex associations between food allergens we propose MBA as a tool to unearth the hidden relationships between various allergens in food allergy research. Market Basket Analysis is utilized to examine how it can serve as a viable option in assessing coassociations in food allergen research.

Table 3: Market Basket Analysis results showcasing associations between food items. The table details Left Hand Side (LHS) and Right Hand Side (RHS) items, along with metrics such as support, confidence, coverage, lift, and the count of occurrences.

**Table 3.** Results for Market Basket Analysis (Arranged by Descending Order of Lift).

LHS	RHS	Support	Confidence	Coverage	Lift	Count
Banana	Avocado	0.04	0.36	0.10	3.18	8
Avocado	Banana	0.04	0.33	0.11	3.18	8
Baked milk*	Unbaked Cow's milk	0.05	1.00	0.05	2.84	11
Soy	Unbaked Cow's milk	0.1	0.96	0.11	2.71	22
Unbaked Cow's milk	Soy	0.1	0.30	0.35	2.71	22
Wheat	Oat	0.02	0.50	0.05	2.63	5
Oat	Wheat	0.02	0.13	0.19	2.63	5
Banana	Rice	0.03	0.32	0.10	1.86	7
Rice	Banana	0.03	0.19	0.17	1.86	7
Oat	Rice	0.06	0.30	0.19	1.75	12
Rice	Oat	0.06	0.33	0.17	1.75	12
Root vegetables	Oat	0.03	0.25	0.11	1.31	6
Oat	Root vegetables	0.03	0.15	0.19	1.31	6
Root vegetables (Potatoes, Sweet Potatoes)	Rice	0.02	0.21	0.11	1.22	5
Avocado	Rice	0.02	0.21	0.11	1.22	5
Rice	Avocado	0.02	0.14	0.17	1.22	5
Rice	Root vegetables	0.02	0.14	0.17	1.22	5
Rice	Unbaked Cow's milk	0.07	0.39	0.17	1.10	14
Unbaked Cow's milk	Rice	0.07	0.19	0.35	1.10	14
Avocado	Unbaked Cow's milk	0.02	0.21	0.11	0.59	5
Egg	Unbaked Cow's milk	0.03	0.19	0.18	0.54	7
Oat	Unbaked Cow's milk	0.03	0.18	0.19	0.50	7

*It is known that individuals allergic to baked milk will be allergic to unbaked milk.^12^ Individuals allergic to baked milk or baked egg are considered a subset of those allergic to raw milk and egg.^13^

#### Key findings

Our analysis revealed distinct patterns of allergen associations in children diagnosed with FPIES. For cow's milk FPIES, a strong link was observed with soy, showing a confidence level of approximately 95.7%. This implies that most individuals with soy FPIES are likely to have concurrent cow's milk FPIES. Other allergens such as avocado, egg, oat, and rice showed lower confidence levels, ranging from approximately 17.5% to 38.9% in their association with cow's milk.

In the case of rice FPIES, banana emerged as the most strongly associated item, with a confidence of 31.8%, indicating that rice FPIES is often considered alongside banana FPIES in our cohort. Oat FPIES also showed a significant association with rice at a 30% confidence level, followed by avocado and sweet potato, both at 20.8%. Additionally, dairy was associated with rice FPIES in nearly 19% of cases.

The analysis of avocado and banana FPIES highlighted a mutual association, with avocado having a 33.3% likelihood of co-occurring with banana FPIES, and vice versa, the confidence for banana co-occurring with avocado FPIES was at 36.4%. Regarding oat FPIES, wheat was found to have a 50% likelihood of coassociation with oat FPIES in our cohort. However, wheat and oat correactivity were only observed in 5 patients and caution should be taken while interpreting these small numbers.

Overall insights from the study underscored some robust associations, notably between banana and avocado, which presented the highest lift value at approximately 3.18, suggesting that this allergen combination is over 3 times more likely to occur together than by chance. Furthermore, the pairing of soy and cow's milk FPIES had the highest count of co-occurrences, with 22 cases noted, underscoring it as a frequent and significant association within our data. These findings contribute valuable insights into the interrelationships of food allergens in FPIES, aiding in better management and understanding of this complex condition.

**Figure 3.**
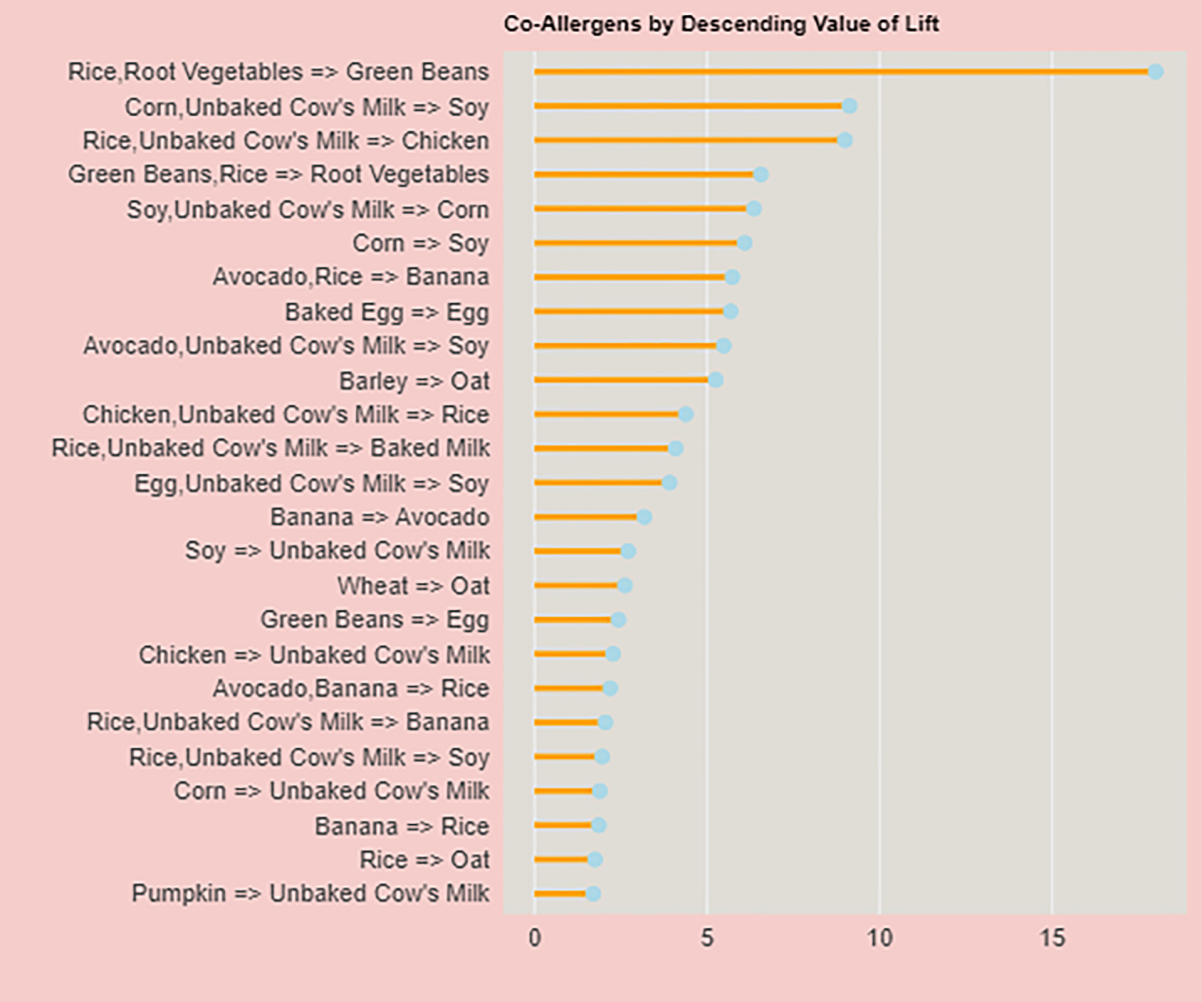
Lift as one of the important determinants of correactivity between food allergies.

In Figure 3 we visualized the top 25 coallergen associations based on the descending values of lift, which is a measure of how much more likely 2 allergens are to occur together than would be expected by chance. Unlike Table 3, which reports values for coallergens reported by 5 or more individuals, Figure 3 focuses on the descending value for lift.

A notable finding was the association between corn and unbaked cow's milk with soy, which demonstrated a lift value of 9.13, a support of 0.02, and a confidence of 1. This implies that in our dataset, patients who had FPIES to both corn and unbaked cow's milk were consistently allergic to soy as well.

Another significant association identified was between avocado and rice with banana, which showed a lift of 5.72, a support of 0.01, and a confidence of 0.6. This indicates that patients allergic to both avocado and rice may have a moderately high likelihood of also being allergic to bananas. This pattern was observed in 4 individuals, suggesting a noteworthy link between these food items.

## Discussion

We conducted this study to elaborate on the use of MBA in FPIES research, a potentially novel method of identifying potential coallergens in FPIES patients. Although MBA is an established method in the retail sector, as far as we know, this is the first instance of its application in the field of food allergy. Our results add to the existing knowledge on FPIES prevalence and triggers, offering useful information for clinicians who manage FPIES patients. The prevalence of FPIES at our center was 0.6%. This is similar to other studies that reported prevalence of 0.14% to 0.7%.^14–17^ The most common foods that triggered FPIES in our population for all 210 patients were cow's milk (35.2%), oats (19%), egg (17.6%), and rice (17.6%). Caubet et al previously reported soy to be an FPIES trigger in 41% of the studied population, while in our population soy was reported to be a trigger in 10.6% of patients. Cow's milk was the most common trigger (44%) in the population examined by Caubet et al,^18^ Gernet (20%), and Lemoine (60.3%).^19,20^ Lemoine et al reported different findings in a French population of children with FPIES, where rice, oat, and soy were rarely or not involved.^20^

We also found that 53.3% of FPIES patients in our population reacted to only one food, while 36.2% reacted to 2 to 3 foods and 10.5% reacted to >3 foods. In a previous study done on 74 individuals at the same institution (with no overlapping individuals) reported 31% with FPIES allergy to one trigger, 34% reporting FPIES allergy to 2 to 3 triggers, and 35% reporting FPIES allergy to more than 3 triggers.^21^ The proportion of responding to multiple triggers in our current FPIES cohort is in line with the findings of Caubet et al,^18^ who reported that 65% of FPIES patients reacted to one food, 26% reacted to 2 foods, and 9% reacted to 3 or more foods. Gernert et al reported a much higher proportion (84% of the individuals) who reported only one FPIES trigger.^19^ Mehr et al and Ruffner et al reported 68% and 42% of their studied population having just one FPIES trigger, respectively.^2,22^ Moreover, we observed a cross-reactivity rate between rice and oats of 30%, which is more or less similar to what Caubet et al found (out of 46 patients reporting FPIES allergy to either oats or soy, 35% reported coreactivity to both).^2,18^ Another notable association that we confirmed is that of cow's milk and soy. In our population, out of the 23 children who were allergic to soy, 22 were allergic to cow's milk (96%). Out of 74 children allergic to cow's milk, 22 were allergic to soy (29.7%). It should be noted that in Blackman et al, which consisted of an FPIES cohort of 74 patients at our center, reported cross reactivity to cow's milk and soy in 53% of the individuals studied.^21^ A similar association was reported by Ruffner (43.5%).^22^ Lower associations were reported by Caubet (21 out of 160 individuals reporting FPIES allergies to cow's milk and soy).^18^

By using MBA, we discovered several strong associations between different food triggers that can inform FPIES management strategies. For example, the strong association we found between soy and cow's milk (confidence: 0.96, lift: 2.71) implies that FPIES patients with a soy trigger may have a higher likelihood of having a coallergy to dairy. Though this association was previously documented, this study is able to quantify the relationship and confirm this relationship. This information could be useful for clinicians when advising patients and/or caretakers on dietary introductions.^23^ Our study adds to the existing research on FPIES, highlighting the need for more studies examining interrelationships between different food allergens.

Wright et al highlighted MBA's rapid data processing, real-time insights, and quantitative metrics in healthcare, while cautioning about its limitations in detecting less common or indirect relationships and the potential to reinforce suboptimal practices.^6^ In this study, the application of MBA to FPIES revealed intricate interrelationships among various food allergens, underscoring the potential of data-driven methods in allergology. MBA approach facilitated a comprehensive analysis of allergen co-occurrences, providing valuable insights into the patterns and probabilities of allergic reactions. Primarily, MBA demonstrated its efficacy in delineating both common and infrequent allergen combinations, a crucial aspect in understanding the complexity of food allergies. For instance, the significant associations identified between allergens such as soy and dairy, and between avocado and banana, highlight the nuanced interplay in allergen coassociation. Furthermore, the quantitative metrics yielded by MBA, including support, confidence, coverage, lift, and count, offered a granular view of these relationships. This level of detail is essential for accurately assessing the likelihood and intensity of allergic reactions, guiding clinical decisions and patient management. Despite these advantages, the application of MBA in food allergy research is not without its challenges. A notable limitation is the potential oversight of rare yet clinically significant allergen associations, a consequence of the method's reliance on the frequency of data occurrences. This highlights the need for cautious interpretation of MBA results, especially when dealing with less common allergens or indirect associations. Moreover, the success of MBA heavily depends upon the quality and completeness of the dataset. Inaccurate or incomplete data can lead to misleading conclusions.

Our study has some limitations. Firstly, the relatively small sample size might limit the generalizability of our findings to the wider population. Despite Houston being one of the most diverse cities in the United States, our FPIES population was predominantly White. The results of this study may not be generalized to low- and middle-income countries (LMICs) which face unique challenges compared to high-income economies.^24,25^ Currently, the literature lacks any reports of FPIES from LMICs. This may be the result of a lack of resources to identify and diagnose these patients or that FPIES is extremely rare or nonexistent in these countries. Future studies with larger sample sizes and more diverse populations would increase the validity of our observations. Future longitudinal studies are recommended to track the evolution of allergenic responses in FPIES patients, providing insights into how these reactions change as children age. Additionally, MBA serves as a tool to assist clinicians in identifying the risk of coassociations between foods. Ultimately, a food challenge must be performed in the clinical setting in order to determine if a food can be tolerated, thus MBA cannot serve as a replacement to assess sensitivity to a food allergen. We suggest creating a multicenter FPIES registry to conduct longitudinal studies on FPIES prevalence, triggers, and outcomes. By pooling data from various centers, we can obtain a larger and more diverse sample size, addressing some of the limitations encountered in our study. Despite these limitations, our findings suggest the usefulness of MBA in identifying patterns of coassociation between different food trigger(s) in FPIES patients. The results support a wider use of MBA in food-allergy data analyses.

In conclusion, MBA offers a framework for exploring the complex landscape of food allergies. However, its application requires careful consideration of its inherent limitations and challenges. Ensuring accurate data collection and mindful interpretation of results are paramount to harnessing the full potential of MBA in advancing our understanding of food allergen interactions and improving patient care strategies in the context of FPIES.

## Supplemental Material

sj-docx-1-hme-10.1177_23333928241264020 - Supplemental material for Applying Market Basket Analysis to Determine Complex Coassociations Among Food Allergens in Children With Food Protein-Induced Enterocolitis Syndrome (FPIES) Supplemental material, sj-docx-1-hme-10.1177_23333928241264020 for Applying Market Basket Analysis to Determine Complex Coassociations Among Food Allergens in Children With Food Protein-Induced Enterocolitis Syndrome (FPIES) by Ankona Banerjee, Kenneth Nobleza, Cynthia Haddad, Joshua Eubanks, Ruchit Rana, Nicholas L. Rider, Lisa Pompeii, Duc Nguyen and Sara Anvari in Health Services Research and Managerial Epidemiology
