# Supplementary material for: Applying Market Basket Analysis to Determine Complex Coassociations Among Food Allergens in Children With Food Protein-Induced Enterocolitis Syndrome (FPIES)
Source: Health Serv Res Manag Epidemiol. 2024 Jul 25;11:23333928241264020. doi: 10.1177/23333928241264020 (PMC11274009; doi:10.1177/23333928241264020)
Supplement: sj-docx-1-hme-10.1177_23333928241264020 - Supplemental material for Applying Market Basket Analysis to Determine Complex Coassociations Among Food Allergens in Children With Food Protein-Induced Enterocolitis Syndrome (FPIES) [file sj-docx-1-hme-10.1177_23333928241264020.docx]

Supplementary Table 1: : Differences in food triggers between the group with one FPIES trigger versus more than one FPIES trigger for all food items reported in our study population.

| Variable | Total, n (%)(95% CI), 95% CI) | One trigger, n (%)( 95% CI) | More than one triggers, n (%)( 95% CI) | p |
| --- | --- | --- | --- | --- |
| n | 210 | 112 | 98 |  |
| Almond (%) | 4 (1.9)(0.6-5.1) | 0 (0.0) | 4 (4.1)(1.3-10.7) | 0.046 |
| Apple (%) | 5 (2.4)(0.8-5.7) | 1 (0.9)(0.04-5.6) | 4 (4.1) (1.3-10.7) | 0.187 |
| Avocado (%) | 24 (11.4)(7.6-16.7) | 5 (4.5)(1.6-10.6) | 19 (19.4)(12.4-28.9) | 0.001 |
| Baked milk (%) | 11 (5.2)(2.8-9.4) | 0 (0.0) | 11 (11.2)(6-19.6) | <0.001 |
| Baked egg (%) | 3 (1.4)(0.4-4.5) | 0 (0.0) | 3 (3.1)(0.8-9.3) | 0.100 |
| Banana (%) | 22 (10.5) | 6 (5.4) | 16 (16.3) | 0.012 |
| Barley (%) | 3 (1.4) (0.4-4.5) | 0 (0.0) | 3 (3.1) )(0.8-9.3) | 0.100 |
| Beef (%) | 3 (1.4) (0.4-4.5) | 1 (0.9) (0.04-5.6) | 2 (2.0) (0.4-7.9) | 0.600 |
| Berries (%) | 2 (1.0)(0.2-3.8) | 0 (0.0) | 2 (2.0) (0.4-7.9) | 0.217 |
| black beans (%) | 2 (1.0)(0.2-3.8) | 0 (0.0) | 2 (2.0) (0.4-7.9) | 0.217 |
| Leafy vegetables | 2 (1.0) (0.2-3.8) | 0 (0.0) | 2 (2.0) (0.4-7.9) | 0.217 |
| Cashew (%) | 2 (1.0) (0.2-3.8) | 1 (0.9) (0.04-5.6) | 1 (1.0)(0.05-6.4) | 1.000 |
| Chicken (%) | 5 (2.4)(0.9-5.7) | 0 (0.0) | 5 (5.1)(1.9-12) | 0.021 |
| Coconut (%) | 1 (0.5) (0.02-3.0) | 0 (0.0) | 1 (1.0) (0.05-6.4) | 0.467 |
| Corn (%) | 6 (2.9)(1.2-6.4) | 0 (0.0) | 6 (6.1)(2.5-13.4) | 0.009 |
| Cow’s milk (%) | 74 (35.2)(28.9-42.1) | 29 (25.9)(18.3-32.2) | 45 (45.9)(35.9-56.3) | 0.004 |
| Egg (%) | 37 (17.6)(12.9-23.6) | 18 (16.1)(10.1-24.5) | 19 (19.4)(12.4-28.9) | 0.59 |
| Stone Fruit (%) | 1 (0.5) (0.02-3.0) | 0 (0.0) | 1 (1.0) (0.05-6.4) | 0.467 |
| Grapes (%) | 1 (0.5) (0.02-3.0) | 0 (0.0) | 1 (1.0) (0.05-6.4) | 0.467 |
| Green beans (%) | 7 (3.3)  (1.5-7.0) | 0 (0.0) | 7 (7.1)(3.2-14.7) | 0.004 |
| Mango (%) | 2 (1.0) (0.02-3.0) | 0 (0.0) | 2 (2.0) (0.4-7.9) | 0.217 |
| Melons (%) | 1 (0.5) (0.02-3.0) | 0 (0.0) | 1 (1.0) (0.05-6.4) | 0.467 |
| Oat (%) | 40 (19.0)(14.1-25.2) | 10 (8.9)(4.6-16.2) | 30 (30.6)(21.9-40.9) | <0.001 |
| Pea (%) | 1 (0.5) (0.02-3.0) | 0 (0.0) | 1 (1.0) (0.05-6.4) | 0.467 |
| Peaches (%) | 1 (0.5) (0.02-3.0) | 0 (0.0) | 1 (1.0) (0.05-6.4) | 0.467 |
| Peanuts (%) | 21 (10.0)(6.4-15.1) | 13 (11.6) (6.6-19.4) | 8 (8.2) (3.8-15.9) | 0.492 |
| Pear (%) | 2 (1.0) (0.02-3.0) | 0 (0.0) | 2 (2.0) (0.4-7.9) | 0.217 |
| plantain (%) | 1 (0.5) (0.02-3.0) | 0 (0.0) | 1 (1.0) (0.05-6.4) | 0.467 |
| Pork (%) | 1 (0.5) (0.02-3.0) | 0 (0.0) | 1 (1.0) (0.05-6.4) | 0.467 |
| Quinoa (%) | 2 (1.0) (0.2-3.8) | 0 (0.0) | 2 (2.0) (0.4-7.9) | 0.217 |
| Scallop (%) | 1 (0.5) (0.02-3.0) | 0 (0.0) | 1 (1.0) (0.05-6.4) | 0.467 |
| Shrimp (%) | 4 (1.9) (0.6-5.1) | 4 (3.6) (1.2-9.4) | 0 (0.0) | 0.125 |
| Sunflower Seeds (%) | 1 (0.5) (0.02-3.0) | 0 (0.0) | 1 (1.0) (0.05-6.4) | 0.467 |
| Tomato (%) | 1 (0.5) (0.02-3.0) | 0 (0.0) | 1 (1.0) (0.05-6.4) | 0.467 |
| Turkey (%) | 1 (0.5) (0.02-3.0) | 0 (0.0) | 1 (1.0) (0.05-6.4) | 0.467 |
| Unknown Fish (%) | 1 (0.5) (0.02-3.0) | 1 (0.9) (0.04-5.6) | 0 (0.0) | 1.000 |
| Unknown (Fishsticks) (%) | 1 (0.5) (0.02-3.0) | 1 (0.9) (0.04-5.6) | 0 (0.0) | 1.000 |
| Root vegetables | 24 (11.4)(7.6-16.7) | 9 (8.0)(4.0-15.1) | 15 (15.3)(9.1-24.3) | 0.128 |
| Pumpkin (%) | 5 (2.4) | 1 (0.9) (0.04-5.6) | 4 (4.1) (1.3-10.7) | 0.187 |
| Chickpea (%) | 3 (1.4) )(0.4-4.5) | 0 (0.0) | 3 (3.1) )(0.8-9.3) | 0.100 |
| Kiwi (%) | 2 (1.0) (0.2-3.8) | 0 (0.0) | 2 (2.0) (0.4-7.9) | 0.217 |
| Rice (%) | 36 (17.1)(12.4-23.1) | 5 (4.5)(1.7-10.6) | 31 (31.6)(22.8-41.9) | <0.001 |
| Soy (%) | 23 (11.0)(7.2-16.2) | 1 (0.9)(0.04-5.6) | 22 (22.4) (14.9-32.2) | <0.001 |
| Wheat (%) | 10 (4.8)(2.4-8.8) | 4 (3.6)(1.2-9.4) | 6 (6.1)(2.5-13.4) | 0.52 |
